# *PREMATURE SENESCENCE LEAF 50* Promotes Heat Stress Tolerance in Rice (*Oryza sativa* L.)

**DOI:** 10.1186/s12284-021-00493-w

**Published:** 2021-06-12

**Authors:** Yan He, Xiaobo Zhang, Yongfeng Shi, Xia Xu, Liangjian Li, Jian-Li Wu

**Affiliations:** State Key Laboratory of Rice Biology, China National Rice Research Institute, Hangzhou, 310006 China

**Keywords:** Rice, Senescence, IMBR, Heat tolerance, Hydrogen peroxide

## Abstract

**Background:**

Heat stress is a major environmental factor that could induce premature leaf senescence in plants. So far, a few rice premature senescent leaf mutants have been reported to involve in heat tolerance.

**Findings:**

We identified a *premature senescence leaf 50* (*psl50*) mutant that exhibited a higher heat susceptibility with decreased survival rate, over-accumulated hydrogen peroxide (H_2_O_2_) content and increased cell death under heat stress compared with the wild-type. The causal gene *PREMATURE SENESCENCE LEAF 50* (*PSL50*) was isolated by using initial map-based resequencing (IMBR) approach, and we found that *PSL50* promoted heat tolerance probably by acting as a modulator of H_2_O_2_ signaling in response to heat stress in rice (*Oryza sativa* L.).

**Conclusions:**

*PSL50* negatively regulates heat-induced premature leaf senescence in rice.

**Supplementary Information:**

The online version contains supplementary material available at 10.1186/s12284-021-00493-w.

## Findings

In plants, premature leaf senescence is one of the major symptoms resulting from heat stress. During heat-induced senescence, leaf cells undergo a series of cellular changes including reactive oxygen species (ROS) accumulation, photosynthetic apparatus impairment and cell death (Cui et al. 2020; Ivanov et al. 2017; Lee et al. 2014). Accordingly, plants have developed complex biochemical regulatory mechanisms to response and adapt to heat stress. For example, Arabidopsis HEAT SHOCK PROTEINS 90 (HSP90) controls the initiation of stomatal cell lineage coupled with stomatal development to adapt to heat-stress by phosphorylating MITOGEN-ACTIVATED PROTEIN KINASEs (MPK3 and MPK6) cascades (Samakovli et al. 2020). In rice *high temperature susceptibility* (*hts*) semi-rolled leaf mutant, abscisic acid acts as a negative regulator of heat stress by mediating energy homeostasis (Li et al. 2020). The plant pectin methylesterases modulate cell wall porosity and have been shown to exhibit structural variation in heat stress response (Wu et al. 2018). However, little is known about the underlying genetic and molecular mechanisms of the connection between leaf premature senescence and heat stress.

**Fig. 1:**
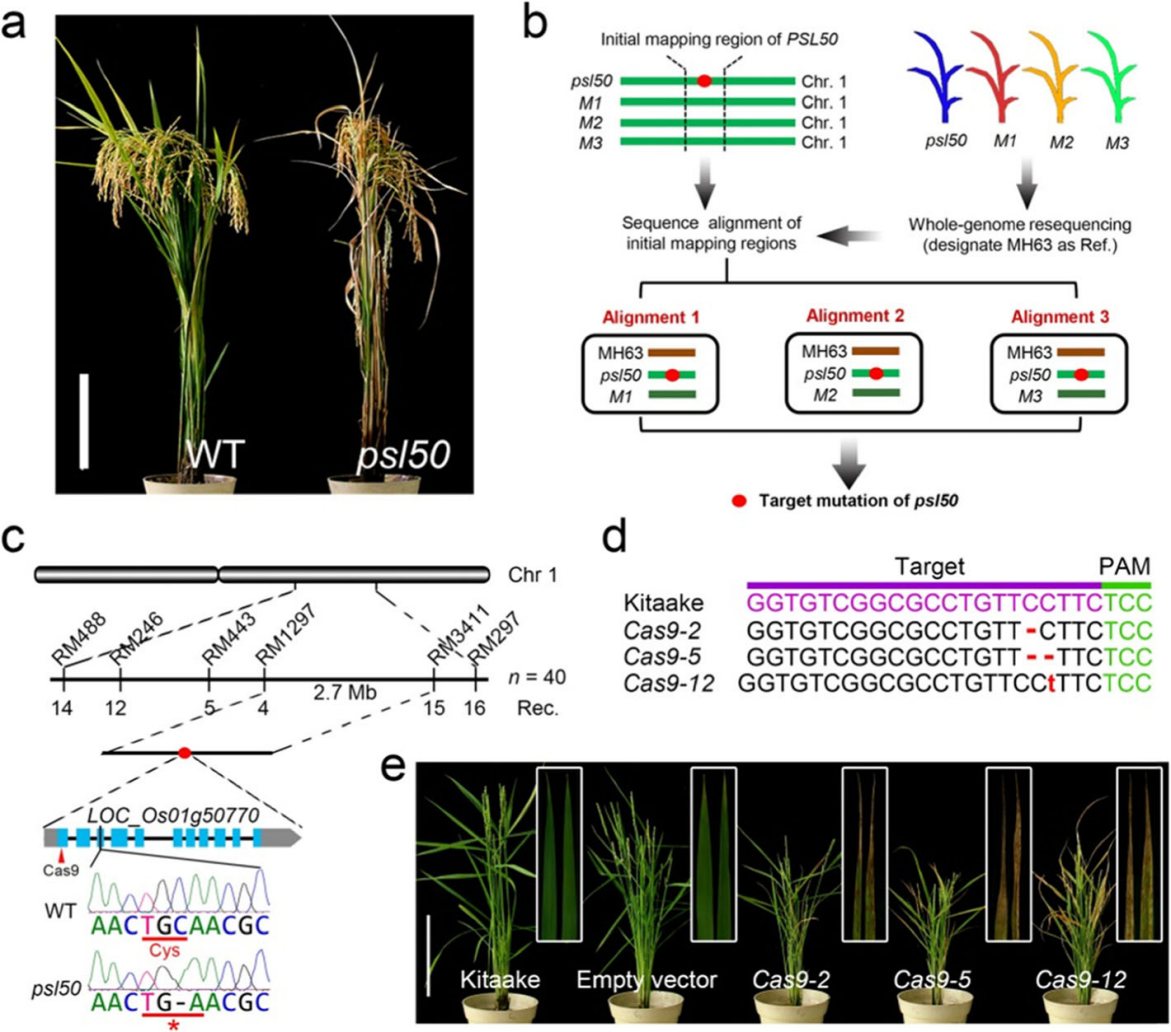
Identification of *PSL50* by IMBR strategy. **a** Phenotype of WT and *psl50* at the mature stage. Scale bar = 20 cm. **b** Identification of target mutation of *psl50* by IMBR strategy. Red dot indicates the causative mutation responsible for the *psl50* phenotype. **c** Initial mapping of *PSL50* and verification of target mutation in *PSL50*. **d** Deletion and insertion mutation at the target site of 1st exon in three representative knockout lines generated by CRISPR/Cas9 in ‘Kitaake’ background. The *Cas9–2*, *Cas9–5* and *Cas9–12* lines are homozygous mutants carrying a 1-bp deletion, a 2-bp deletion and a 1-bp insertion on both homochromosomes, respectively. **e** Phenotype of three representative T_0_ knockout lines at the heading stage. Insets display magnified views of flag leaves. Empty vector represents transgenic Kitaake plant transformed with CRISPR/Cas9 empty vector. Scale bar = 20 cm

We previously characterized physio-biochemically an ethyl methane sulfonate (EMS) induced Zhongjian 100 (wild type, WT) mutant, *premature senescence leaf 50* (*psl50*), which displayed severe premature senescent phenotype at the grain-filling stage (Fig. 1a) (He et al. 2018).

To rapidly isolate the causal gene *PSL50* responsible for the premature senescent phenotype, we designed an initial map-based resequencing (IMBR) strategy to locate the candidate gene by combining initial mapping and whole-genome resequencing (Fig. 1b). To perform IMBR, *PSL50* was firstly mapped to a large region covering several megabit nucleotides after the initial mapping using a small F_2_ population. Secondly, *psl50* and three randomly chosen mutants (*M1*, *M2* and *M3*) from the same mutant bank were sampled for whole-genome resequencing. Lastly, the sequences of the target initial mapping region from the four lines were aligned and compared with the reference genome of indica rice Minghui 63 (MH63, http://rice.hzau.edu.cn/cgi-bin/gb2/gbrowse/MH63RS2/) (Zhang et al. 2016) to detect SNP and/or InDel variations. It is not necessary to sequence WT because the sequences of four mutant lines can be used as control or biological replicates to lower/eliminate sequencing errors.

As a proof of concept, a total of 178 polymorphic simple sequence repeat (SSR) markers evenly distributed over 12 chromosomes between parental line *psl50* and japonica line 80A90YR72 were used for linkage analysis of two bulked segregant analysis (BSA) DNA pools derived from the wild-type and mutant type F_2_ progenies from the cross *psl50*/80A90YR72. The results showed that three SSR markers RM297, RM443 and RM488 were co-segregated with the mutation. Further mapping indicated that *PSL50* localized to a 2.7-Mb genomic region between RM3411 and RM1297 at the long arm of chromosome 1 by using 40 *psl50-*type F_2_ individuals derived from the cross *psl50*/80A90YR72 (Fig. 1c). Sequence alignments of the 2.7-Mb region showed that there were 155, 162 and 158 SNPs/InDels between *psl50/M1*, *psl50*/*M2* and *psl50/M3*, respectively (Additional file 2: Table S1-S3). Obviously, most SNPs/InDels presented two types of genotype between *psl50* and the other three mutants, we cannot consider them as the true SNPs/InDels. Only the site(s) showing a single genotype at all allele depth was considered as true SNPs/InDels. As shown in Table S1, S2, S3, only the position at 28,281,174 within the third exon of *LOC_Os01g50770* exhibited the nucleotide substitution from GC to G with single genotype in allele depth among all the sequence alignments between *psl50* (G/G with 0,24 allele depth) and the other three mutants (GC/GC with 10,0 allele depth for *M1*, 14,0 for *M2* and 18,0 for *M3*, respectively). These results revealed that the variation from GC to G at the position 28,281,174 was a true 1-bp deletion mutation in *LOC_Os01g50770* (Table 1; Fig. 1c).

**Table 1:** Nucleotide comparison of target mutation site between *psl50* and the other three mutants

Material	Position	Genotype	Mutant depth	Mutant allele depth	Mutation type
*psl50*	28,281,174	G	10	10,0	GC to G (frameshift)
*M1*	28,281,174	GC	14	14,0	GC (wild-type)
*M2*	28,281,174	GC	18	18,0	GC (wild-type)
*M3*	28,281,174	GC	24	24,0	GC (wild-type)
MH63	28,281,174	GC			GC

We further performed sequencing on polymerase chain reaction (PCR) products to confirm the 1-bp deletion mutation which leads to a premature stop codon (Fig. 1c; Additional file 1: Figure S1a). *LOC_Os01g50770* is predicted to encode a clathrin-associated adaptor protein complex 1 medium subunit μ1 (AP1M1). The mutation type *LOC_Os01g50770* is predicted to encode a truncated protein (ΔPSL50) lacking the AP-1 complex subunit μ N-terminal domain, and the functional domain deletion could be observed visually by modeling the three-dimensional protein structures of PSL50 and ΔPSL50 (Additional file 1: Figure S1b, c). To quickly demonstrate whether the mutation of *LOC_Os01g50770* was responsible for premature leaf senescence in *psl50*, we transformed a CRISPR/Cas9 construct targeting the first exon of *LOC_Os01g50770* into a japonica rice variety Kitaake (Fig. 1d). Expectedly, all 13 knockout homozygous lines of T_0_ transgenic plants showed premature senescence leaf phenotype similar to *psl50* (Fig. 1e). These results confirmed that *LOC_Os01g50770* was *PSL50*, and the mutation of *PSL50* caused the premature senescence leaf phenotype in *psl50.* Furthermore, we also successfully identified other true mutations in the three control mutants (data not shown), indicating the feasibility of IMBR strategy in rapid gene isolation.

**Fig. 2:**
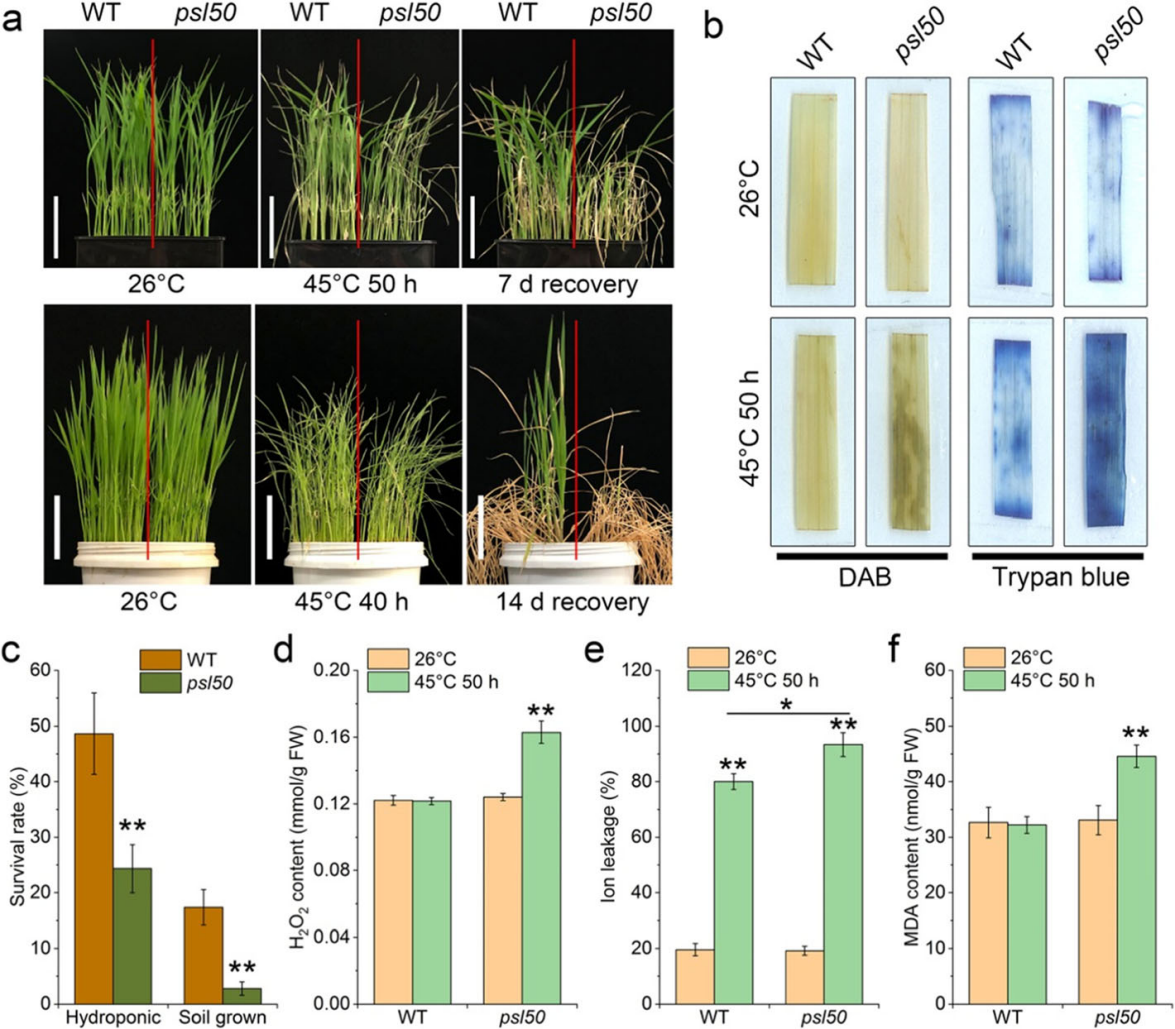
Effects of heat stress on *psl50* and WT at the seedling stage. **a** Phenotypes of WT and *psl50* seedlings under heat stress. Seedlings were hydroponically (upper row) or soil grown (lower row) at 26 °C for 12 d and then treated at 45 °C for 50 h or 40 h followed a recovery at 26 °C. Scale bars = 5 cm. **b** DAB staining for H_2_O_2_ accumulation detection and trypan blue staining for cell death detection in the top 2nd leaves of hydroponical seedlings before and after heat treatment. **c** Survival rate of hydroponic WT and *psl50* seedlings shown in **a**. **d-f** H_2_O_2_ content, ion leakage rate and MDA content in hydroponic WT and *psl50* seedlings before and after heat treatment. Data are means ± SD (*n* = 3). Asterisks indicate significant difference by Student’s *t* test (**P < 0.01 and *P < 0.05)

Many senescence-associated mutants have been identified in various plant species (Liang et al. 2014; Zhao et al. 2016; Shim et al. 2019), while most of the studies focused on the interpretation of senescence-related genetic mechanisms. To explore the association between environmental factors and premature leaf senescence, we carried out a heat treatment on *psl50* at the seedling stage. Under normal growth conditions at 26 °C, no any overt abnormalities or defects were observed in *psl50* compared with WT. However, when 12-day-old seedlings were subjected to heat treatment at 45 °C under hydroponics and soil growth conditions, *psl50* was more susceptible to heat stress and showed lower survival rate than those of WT (Fig. 2a, c). Hydrogen peroxide (H_2_O_2_) over-accumulation is shown to induce plant cell death (He et al. 2020; Sathe et al. 2019). We hence detected H_2_O_2_ accumulation and cell death of *psl50* seedlings with and without heat treatment by using 3,3′-diaminobenzidine (DAB) and trypan blue staining. The results showed that *psl50* had similar level of H_2_O_2_ accumulation and cell death before heat treatment at 26 °C, whereas *psl50* had higher levels of H_2_O_2_ accumulation and cell death after heat treatment at 45 °C compared with WT (Fig. 2b, d). Although ion leakage rate was dramatically increased both in WT and *psl50* after heat treatment, *psl50* showed significantly increased ion leakage rate compared to WT (Fig. 2e). As another indicator of cell membrane damage, the malonaldehyde (MDA) content was similar between *psl50* and WT before heat treatment, while obviously increased in *psl50* after heat treatment (Fig. 2f). These results indicated that *PSL50* could positively contribute to heat tolerance, while dysfunction of *PSL50* in *psl50* resulted in higher heat stress susceptibility associated with cell membrane damage and H_2_O_2_-induced cell death. In addition, the H_2_O_2_ content was similar in green leaves of WT and *psl50*, while prominently increased in premature senescent leaves of *psl50* (Additional file 1: Figure S2). Considering the rapid premature leaf senescence occurred in *psl50* at the grain-filling stage which often accompanied with higher temperatures in natural conditions, we speculated that high temperatures might act as an inducer for premature leaf senescence in *psl50*, involving H_2_O_2_ signaling response pathway.

**Fig. 3:**
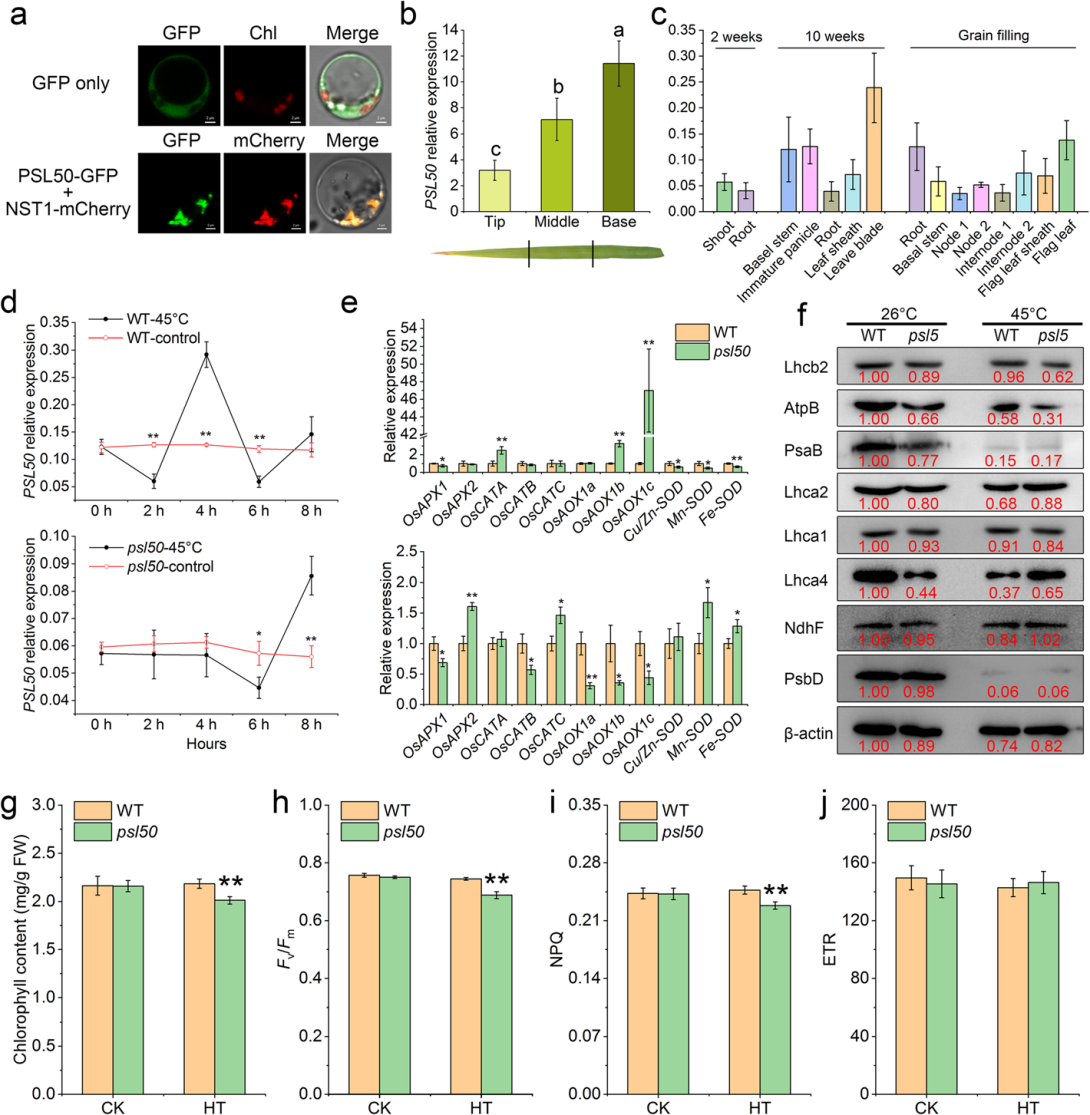
Subcellular localization and expression analysis of *PSL50*. **a** GFP signals in rice protoplasts. NST1-mCherry was used as a rice protoplast Golgi-tagged marker. **b** *PSL50* expression in different parts of a flag leaf. Different letters indicate significant differences according to one-way ANOVA and Duncan’s test (*P* < 0.05). Data are mean ± SD (*n* = 3). **c** Relative expression levels of *PSL50* in various organs at different growth stages. Data are presented as mean ± SD (*n* = 3). **d** Expression analysis of *PSL50* in hydroponic WT and *psl50* seedlings under heat stress conditions. Data are mean ± SD (*n* = 3). **e** Expression analysis of ROS scavenging system-related genes in hydroponic WT and *psl50* seedlings at 26 °C (upper) and 45 °C (lower) for 12 h. Data are mean ± SD (*n* = 3). Rice *UBIQUITIN* (*LOC_Os03g13170*) was used as an internal control. **f** Levels of thylakoid membrane proteins detected in total proteins from top second leaves of 12 d WT and *psl50* hydroponic seedlings at 26 °C and 45 °C for 50 h. Image J was used for the quantification of immunoblot results and these experiments were repeated at least twice with similar results. **g** Leaf chlorophyll content of hydroponic WT and *psl50* seedlings before and after heat treatment. Data are means ± SD (*n* = 3). **h-j** Changes of photochemical efficiency of PSII (*F*_v_/*F*_m_), non-photochemical fluorescence quenching (NPQ) and relative PSII electron transport rate (ETR) in WT and *psl50* under heat treatment. The data for heat treatment in **g-h** were collected after a 7-d recovery at 26 °C. Seedlings were hydroponically grown at 26 °C for 12 d and then treated at 45 °C for 50 h following a 7-d recovery at 26 °C. CK, normal growth condition at 26 °C; HT, heat treatment at 45 °C followed by recovery at 26 °C for 7 d. Data are means ± SD (*n* = 5). Asterisks indicate significant difference by Student’s *t* test (**P < 0.01 and *P < 0.05)

*PSL50* is likely allelic to *SPOTTED LEAF 28* (*SPL28*) in rice, which is involved in the regulation of vesicular trafficking, and the dysfunction of *SPL28* results in the formation of hypersensitive response (HR)-like lesions, leading to the initiation of leaf senescence (Qiao et al. 2010). To detect the subcellular location of PSL50, we fused the green fluorescent protein (GFP) to the C-terminus of PSL50 driven by the CaMV35S promoter to create the PSL50::GFP fusion protein. The PSL50::GFP fluorescent signals were co-localized with NST1::mCherry, a Golgi-tagged marker (Zhang et al. 2011), indicating that PSL50 localized to the Golgi apparatus (Fig. 3a), and this result was consistent with the subcellular localization of SPL28 in onion epidermal cells (Qiao et al. 2010). The expression of *PSL50* was detectable in all different rice organs at different developmental stages, and its expression increased gradually from the top to the base of a fully expanded flag leaf (Fig. 3b, c). In addition, we examined *PSL50* expression in different leaves at the mature stage, and found that *PSL50* transcripts were higher in younger, greener leaves than those of older and senescing leaves (Additional file 1: Figure S3). The results suggested that *PSL50* was widely expressed and acted as a negative regulator for natural rice leaf senescence. To explore the roles of *PSL50* in heat tolerance, we further investigated the kinetic mRNA level alterations of *PSL50* in WT and *psl50* under heat stress conditions. In WT, *PSL50* mRNA levels decreased by 0.4-fold after 2 h heat treatment, and increased rapidly by 2.4-fold after 4 h heat treatment, whereas in *psl50*, a similar variation trend of *PSL50* mRNA levels emerged after 6 h heat treatment (Fig. 3d). The results indicated that *PSL50* transcription was induced by heat stress and the delayed expression of non-functional *PSL50* in *psl50* may be resulted from the loss of PSL50 function.

We also detected the transcripts of ROS-scavenging system associated genes, including ascorbate peroxidase (APX) genes *OsAPX1*and *OsAPX2* (Bonifacio et al. 2016), catalase genes *OsCATA*, *OsCATB* and *OsCATC* (Lin et al. 2012; Ye et al. 2011), alternative oxidase (AOX) genes *OsAOX1a*, *OsAOX1b* and *OsAOX1c* (Fujii and Toriyama 2008; Saika et al. 2002), and superoxide dismutase (SOD) genes *Cu/Zn-SOD*, *Mn-SOD* and *Fe-SOD* (Wang et al. 2016; Guan et al. 2017). APX catalyzes the reduction of H_2_O_2_ into water, and *OsAPX1* was down-regulated in *psl50* under normal growth and heat stress conditions (Fig. 3e). Under normal growth conditions, the reduced level of *OsAPX1* and normal level of *OsAPX2* in *psl50* might be enough to maintain the balance of H_2_O_2_ production and scavenging. Whereas in case of heat stress conditions, the ROS burst was highly induced and the up-regulation of *OsAPX2* in *psl50* might partially compensate for the down-regulated *OsAPX1*(Figs. 2d, 3e). Under normal growth conditions, *OsCATA* was up-regulated in *psl50* while the expressions of *OsCATB/C* were similar to WT, in contrast, the expression of *OsCATB* was highly down-regulated while the expression of *OsCATC* was apparently up-regulated in *psl50* compared with WT under heat stress (Fig. 3e). We speculated that a similar compensation mechanism might occur among CAT isozyme genes (Fig. 3e). Compared to the normal growth conditions at 26 °C, we also found that heat stress restrained the expression of *OsAOX1a*, *OsAOX1b* and *OsAOX1c*, while improved the expression of *Mn-SOD* and *Fe-SOD* in *psl50* (Fig. 3e). AOX is a mitochondrial respiratory oxidase playing important roles in intracellular oxygen molecule scavenging while SOD catalyzes the superoxide anion to H_2_O_2_ (Baurain et al. 2003; Asada 2006). Therefore, the increased transcriptional level of SOD genes and decreased transcriptional level of AOX genes would facilitate the ROS accumulation in *psl50* under heat stress, which likely confers the higher heat susceptibility of *psl50*.

In plants, ROS are mainly produced during the light reaction of photosystem II (PSII) and PSI complex at thylakoids, and PSII is the most thermolabile photosynthetic complex (Asada 2006; Chen et al. 2017). Thus, immunoblot was performed to test whether the major components of thylakoid membrane complexes were impaired under heat stress. As the core subunits of PSII and PSI complexes, PsaB and PsbD were dramatically decreased in both WT and *psl50* after 45 °C heat treatment (Fig. 3f), indicating that heat stress commonly causes severe thermal damage not only to PSII but also PSI. Unlike the decreased levels of LHCII type II chlorophyll *a*/*b*-binding protein Lhcb2 and ATP synthase *β*-subunit (AtpB) in *psl50* after heat treatment, the accumulations of light-harvesting antenna of PSI (LHCI) chlorophyll *a*/*b*-binding proteins Lhca1, was not obviously affected by heat treatment, similarly, the retained Lhca2 and Lhca4 protein levels in *psl50* were near to the WT levels (at 26 °C) after heat treatment (Fig. 3f). Compared with WT, the non-photochemical quenching (NPQ) value of *psl50* was lower under heat treatment, suggesting that heat stress impaired the NPQ process for heat dissipation which serves to prevent damage to PSII, and the decreased PSII antenna Lhcb2 protein level in *psl50* after heat treatment might associate with the weaker NPQ capacity of *psl50* (Fig. 3f, i). Meanwhile, comparable NAD(P)H dehydrogenase subunit 5 (NdhF) levels and relative PSII electron transport rate (ETR) in WT and *psl50* indicated that electron transfer was normal under heat treatment (Fig. 3f, j). To further investigate the harmful effects of *PSL50* disruption on the photosynthetic capacity, we then measured the maximum quantum efficiency of PSII photochemistry (*F*v/*F*m). In agreement with the *psl50* heat-sensitive phenotype, the values of *F*v/*F*m and chlorophyll content were also lower in *psl50* than those of WT under heat stress (Fig. 3g, h). In addition, we determined whether light intensity affects the heat susceptibility of *psl50*. Intriguingly, it was showed that the heat tolerance of both WT and *psl50* plants was obviously facilitated by high light intensity (HL) (Additional file 1: Figure S4a). However, under heat treatment (HT) with normal light intensity (NL), the survival rate of *psl50* was significantly lower than WT after a 7-d recovery, and the *F*v/*F*m was even not detectable in *psl50* (Additional file 1: Figure S4b, c). These results suggested that the higher heat susceptibility of *psl50* may be the consequence of impaired photosynthesis under NL and HT conditions. While under HL and HT conditions, the photosynthetic potential productivity of plants was motivated by HL, which ultimately improved the heat tolerance of both WT and *psl50* and even eliminated the heat tolerance disparity between WT and *psl50*. Plants frequently encounter a combination of two or multiple abiotic stresses in nature at a time. Though combined abiotic stresses generally have greater negative effects on plants than that of single type abiotic stress, previous studies also have shown that some abiotic stresses could actually enhance plant tolerance to another abiotic stress (Choudhury et al. 2017; Miller et al. 2010), and plants are able to integrate two different stress-specific systemic signals (induced by light or/and heat) to improve its acclimation in coordinating different transcriptional responses, such as ROS- and salicylic acid (SA)-transcript variations (Zandalinas et al. 2020). In summary, these data demonstrated that PSL50 is essential for the heat tolerance regulation of rice plants by maintaining the stability of photosynthetic system under heat stress, and the high light intensity can enhance the heat tolerance of both WT and *psl50*.

Taken together, we successfully identified and isolated rice *PSL50* by using the IMBR strategy. Furthermore, we found that PSL50 played important roles both in rice premature leaf senescence and heat stress response, involving in the regulations of H_2_O_2_ accumulation and photosynthetic adaption. This study would facilitate studies on functions of PSL50 in heat stress-induced premature leaf senescence.

## Supplementary Information

**Additional file 1: Figure S1.** Mutation analysis of PSL50. **Figure S2.** Leaf phenotypes and H_2_O_2_ content of WT and *psl50* at 40 d after transplanting. **Figure S3.** *PSL50* expression in different leaves at the mature stage. **Figure S4.** Effects of light intensity on WT and *psl50* seedlings under heat stress.

**Additional file 2: Table S1.** INDEL and SNP between *psl50* and mutant 1. **Table S2.** INDEL and SNP between *psl50* and mutant 2. **Table S3.** INDEL and SNP between *psl50* and mutant 3. **Table S4.** List of primers used in this study.

**Additional file 3.** Materials and Methods (Ma et al. 2015; Yu et al. 2020).

## Data Availability

All data supporting the conclusions of this article are provided within the article (and its additional files).
