# Correction to: PREMATURE SENESCENCE LEAF 50 Promotes Heat Stress Tolerance in Rice (*Oryza sativa* L.)

**DOI:** 10.1186/s12284-021-00506-8

**Published:** 2021-07-05

**Authors:** Yan He, Xiaobo Zhang, Yongfeng Shi, Xia Xu, Liangjian Li, Jian-Li Wu

**Affiliations:** State Key Laboratory of Rice Biology, China National Rice Research Institute, Hangzhou, 310006 China

**Correction to: Rice 14, 53 (2021)**

**https://doi.org/10.1186/s12284-021-00493-w**

It was highlighted that in the original article (He et al. 2021) Additional file 1 was incomplete and missing Figure s4. This Correction article shows the complete Additional file 1. The original article has been updated.

## Supplementary Information

**Additional file 1: Figure S1.** Mutation analysis of PSL50. **A**. Amino acid sequence aligment of PSL50 between WT and *psl50* mutant. **B**. Diagrams of the wild-type PSL50 and mutant PSL50 (ΔPSL50). **C**. Deletion of functional domains shown by modeling the three-dimensional protein structures of wild-type PSL50 and ΔPSL50. The three-dimensional model structures were predicted using Swiss-model (https://swissmodel.expasy.org/interactive). **Figure S2.** Leaf phenotypes and H_2_O_2_ content of wild-type and *psl50* at 40 d after transplanting. L1-L4 represent four leaves from top to bottom, respectively. Data are means ± SD (*n* = 3), *P < 0.05 by Student’s *t* test. **Figure S3.** *PSL50* expression in different leaves at the mature stage. **a** Phenotypes of different leaves at the mature stage. L1-L5 represent five leaves from top to bottom, respectively. **b** *PSL50* expression in different leaves shown in **a**. Data are means ± SD (*n* = 3). **Figure S4.** Effects of light intensity on WT and *psl50* seedlings under heat stress. **a** Phenotypes of WT and *psl50* seedlings under heat stress and different light intensity. NL, normal light intensity (200 μmol m^-2^ s^-1^); HL, High light intensity (500 *μ*mol m^-2^ s^-1^); HT, heat stress at 45°C. 2-week-old hydroponic plants at 26°C with 14 h light/10 h dark cycles (200 *μ*mol m^-2^ s^-1^) were used for the treatment. Scale bars = 5 cm. **b** Photochemical efficiency of PSII (*F*_v_/*F*_m_ ) of WT and *psl50* plants shown in **a**. ND, not detected. Data are means ± SD (*n* = 5). **c** Survival rate of WT and *psl50* plants shown in **a** following a 7 d recovery at 26°C with 14 h light/10 h dark cycles (200 μmol m^-2^ s^-1^). Data are means ± SD for three biological replicates (*n* = 48 for each replicate). Asterisks indicate significant difference by Student’s *t* test (*P < 0.05).
