# Supplementary material for: PREMATURE SENESCENCE LEAF 50 Promotes Heat Stress Tolerance in Rice (Oryza sativa L.)
Source: Rice (N Y). 2021 Jun 12;14:53. doi: 10.1186/s12284-021-00493-w (PMC8197683; doi:10.1186/s12284-021-00493-w)
Supplement: Supplementary file 3 — Additional file 3. Materials and Methods (Ma et al. 2015; Yu et al. 2020). [file 12284_2021_493_MOESM3_ESM.docx]

Materials and Methods

Plant materials and growth conditions

The *psl50*, mutant1 (*M1*), mutant2 (*M2*), mutant3 (*M3*) were obtained from an EMS-induced indica rice accession Zhongjian100 (wild-type, WT) mutant bank. The four mutants, wild-type and F_2_ individual plants from the cross *psl50*/80A90YR72 were grown in the paddy field at the China National Rice Research Institute (CNRRI). For the heat treatment, plants were cultured respectively with soil and hydroponically cultured with Yoshida rice nutrient salt mixture (Coolaber, NSP1040) after germination in a growth chamber at 26°C or 45°C, with 14 h light/10 h dark cycle. To test the effects of light intensity on the heat susceptibility of *psl50*, different light intensities (200 *μ*mol m^-2^ s^-1^ or 500 *μ*mol m^-2^ s^-1^) were given during the heat treatments.

Initial map-based resequencing (IMBR) of *PSL50*

Firstly, equal amounts of leaf blades from each of ten wild-type plants and ten *psl50*-type individual F_2_ plants derived from *psl50*/80A90YR72 were collected for DNA extraction to form a wild-type (WT) DNA pool and a *psl50*-type DNA pool, respectively. These two DNA pools were subjected to preliminary linkage analysis of the mutation by genotyping 178 polymorphic simple sequence repeat (SSR) markers evenly covering 12 chromosomes. Subsequently, 40 *psl50*-type individual F_2_ plants were genotyped to determine the primary physical location of *PSL50*. Approximately 5 mg fresh leaves from each of the four mutants (*psl50*, *M1*, *M2*, and *M3*) were collected to extract DNA (QIAGEN, Lot No. 145034200), and the DNAs were used for whole-genome resequencing at Majorbio company (Shanghai, China). The sequencing alignments were conducted on the cloud platform of Majorbio company (https://www.i-sanger.com/).

PSL50-GFP and CRISPR/Cas9 vector construction

The full CDS of *PSL50* were amplified from WT cDNA and cloned into pAN580 (GFP) vector (digested with *Spe*I restriction enzyme) with Gibson assembly strategy, and the GFP fluorescence signal was detected using Zeiss lsm710 confocal laser scanning microscope (Carl Zeiss, Inc., Jena, Germany). The CRISPR/Cas9 construct of *PSL50* was generated according to a previous report (Ma et al. 2015) . The genetic transformation was conducted by using embryogenic calli induced from the rice cultivar ‘Kitaake’ through Agrobacterium-mediated transformation method. Primers used in the study are listed in Table S1.

Measurements of MDA, H_2_O_2_ content and ion leakage rate

The contents of MDA and H_2_O_2_ were determined using the kits following the manufacturer’s instructions (Nanjing Jiancheng Bioengineering Institute). For the ion leakage analysis, Leaves were cut into 0.5-cm pieces and 50 mg was weighed out precisely, then placed into 20 mL deionized water in a 50 mL tube, followed by vacuum treatment for 10 min, and incubated in a shaker with 100 rpm at 28°C for 30 min. The membrane ion leakage (value A) was measured using a DDS-307A conductivity meter (LeiCi, Hangzhou, China). Then, the samples were incubated in boiling water for 5 min to thoroughly release electrolytes. After the samples were cooled down to the room temperature, the final membrane ion leakage (value B) was measured. The value of membrane ion leakage was calculated by the formula A/B × 100%.

**Measurements of chlorophyll content and chlorophyll fluorescence parameters**

Chlorophyll content was determined by measuring the A_652_ using a SpectraMax i3x multi-mode microplate reader (Molecular Devices) as described previously (He et al. 2018). The chlorophyll fluorescence parameters were collected using a portable chlorophyll fluorescence spectrometer (PAM-2500 chlorophyll fluorescence system; Heinz Walz, Effeltrich, Germany) (Yu et al. 2020).

DAB and trypan blue staining

H_2_O_2_ accumulation and cell death were detected by DAB and trypan blue staining as described previously (He et al. 2018).

Real-time fluorescent quantitative PCR

Total RNA was extracted using a NucleoZOL Reagent Kit (MACHEREY-NAGEL, Düren, Germany) according to the manufacturer’s protocol. RNA was reverse-transcribed using the ReverTra Ace qPCR RT Master Mix with genomic DNA (gDNA) Remover Kit (Toyobo, Osaka, Japan). Real-time fluorescent quantitative PCR (qRT-PCR) was carried out using the FastStar Essential DNA Green Master Kit (Roche, Basel, Switzerland) and performed on a Thermal Cycle Dice Real Time System (Takara, Kusatsu, Japan). For *PSL50* expression analysis, different parts of fully expanded flag leaves at the heading stage and different leaves of WT plants at the mature stage were sampled for RNA isolation. Rice *UBIQUITIN* (*LOC_Os03g13170*) was used as an internal control. Primers used in the study are listed in Table S4.

Protein extraction and immunoblot analysis

Leaf tissues (100 mg) were grounded in liquid nitrogen and placed into 600 µL extraction buffer (0.4 mM Tris-HCl, pH 7.5, 5 mM NaCl, 6.25 µM MgCl_2_ , 10 µM EDTA, 10 µM DL-dithiothreitol, 1% Triton X-100, 2% protease inhibitor) in a 2 mL tube, then incubated in a shaker at 80 rpm, 4°C for 30 min. Homogenates were centrifuged at 4°C with 10,000 *g* for 20 min, and the total supernatant proteins of wild-type and *psl50* were quantified into the same concentration by a BCA Protein Assay Kit (TIANGEN, Beijing, China). The quantified total proteins were denatured at 95°C for 10 min. Total proteins (10µL) of wild-type and *psl50* were subjected to 12% (w/v) polyacrylamide sodium dodecyl sulfate-polyacrylamide gel electrophoresis (SDS-PAGE) and the resolved proteins were transferred onto a PVDF membrane. Antibodies used for immunoblot analysis were purchased from Agrisera (Vännäs, Sweden).
