# Supplementary material for: Correction to: PREMATURE SENESCENCE LEAF 50 Promotes Heat Stress Tolerance in Rice (Oryza sativa L.)
Source: Rice (N Y). 2021 Jul 5;14:63. doi: 10.1186/s12284-021-00506-8 (PMC8257812; doi:10.1186/s12284-021-00506-8)
Supplement: Supplementary file 1 — Additional file 1: Figure S1. Mutation analysis of PSL50. A. Amino acid sequence aligment of PSL50 between WT and psl50 mutant. B. Diagrams of the wild-type PSL50 and mutant PSL50 (ΔPSL50). C. Deletion of functional domains shown by modeling the three-dimensional protein structures of wild-type PSL50 and ΔPSL50. The three-dimensional model structures were predicted using Swiss-model (https://swissmodel.expasy.org/interactive). Figure S2. Leaf phenotypes and H2O2 content of wild-type and psl50 at 40 d after transplanting. L1-L4 represent four leaves from top to bottom, respectively. Data are means ± SD (n = 3), *P < 0.05 by Student’s t test. Figure S3. PSL50 expression in different leaves at the mature stage. a Phenotypes of different leaves at the mature stage. L1-L5 represent five leaves from top to bottom, respectively. b PSL50 expression in different leaves shown in a. Data are means ± SD (n = 3). Figure S4. Effects of light intensity on WT and psl50 seedlings under heat stress. a Phenotypes of WT and psl50 seedlings under heat stress and different light intensity. NL, normal light intensity (200 μmol m-2 s-1); HL, High light intensity (500 μmol m-2 s-1); HT, heat stress at 45°C. 2-week-old hydroponic plants at 26°C with 14 h light/10 h dark cycles (200 μmol m-2 s-1) were used for the treatment. Scale bars = 5 cm. b Photochemical efficiency of PSII (Fv/Fm ) of WT and psl50 plants shown in a. ND, not detected. Data are means ± SD (n = 5). c Survival rate of WT and psl50 plants shown in a following a 7 d recovery at 26°C with 14 h light/10 h dark cycles (200 μmol m-2 s-1). Data are means ± SD for three biological replicates (n = 48 for each replicate). Asterisks indicate significant difference by Student’s t test (*P < 0.05). [file 12284_2021_506_MOESM1_ESM.zip › Figure S1.docx]

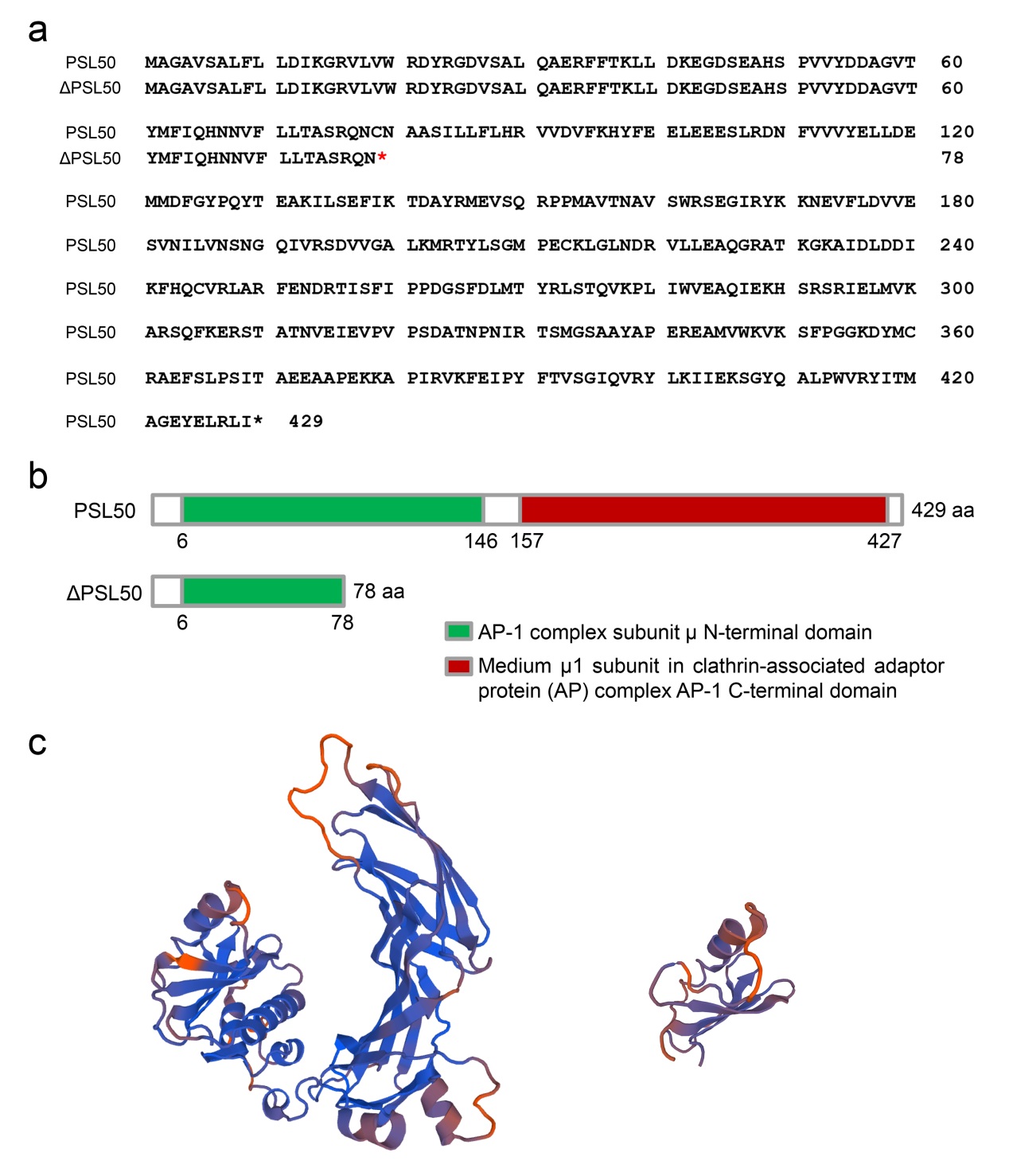


**Fig. S1.** Mutation analysis of PSL50. **A**. Amino acid sequence aligment of PSL50 between WT and *psl50* mutant. **B**. Diagrams of the wild-type PSL50 and mutant PSL50 (ΔPSL50). **C**. Deletion of functional domains shown by modeling the three-dimensional protein structures of wild-type PSL50 and ΔPSL50. The three-dimensional model structures were predicted using Swiss-model (<https://swissmodel.expasy.org/interactive>).
